# Ewing Sarcoma/Primitive Neuroectodermal Tumor of the Kidney: Two Unusual Presentations of a Rare Tumor

**DOI:** 10.1155/2012/190581

**Published:** 2012-01-24

**Authors:** E. C. Castro, A. V. Parwani

**Affiliations:** ^1^Department of Pathology, University of Pittsburgh Medical Center, A71 Scaife Hall, 3550 Terrace Street, Pittsburgh, PA 15261, USA; ^2^Department of Pathology, University of Pittsburgh Medical Center, UPMC Shadyside, 5230 Centre Avenue, WG 02.10, Pittsburgh, PA 15232, USA

## Abstract

Only few cases of primary renal Ewing's sarcoma have been reported in the literature to date. We present here two cases of renal ES/PNET with an uncanny presentation. The first case was discovered after the patient presented clinically with irradiating flank pain, mimicking the pain related with kidney stones. The second case had clinical presentation of pulmonary thromboembolism after the patient was involved in an automobilist accident. The tumors were mainly composed of small blue cells which by immunohistochemical were positive for neural markers, and FISH revealed the translocation 22q12 for the EWSR1 gene. The diagnosis of renal primitive neuroectodermal tumor/EWING tumor is very rare and usually involves several different diagnostic techniques. The differential diagnosis is usually broad with frequent overlapping features between the entities. The cases presented in this paper illustrated the difficulties with which routine anatomical pathologist is faced when dealing with rare renal poorly differentiated neoplasm in adults.

## 1. Introduction

Primitive neuroectodermal tumors (PNETs) are small, round-cell tumors of neural crest origin classically found in the central nervous system (CNS) but more recently characterized in the periphery [1]. Peripherally located PNETs (pPNETs) are members of the Ewing's sarcoma family of tumors (EFTs). It is the second most common primary tumor of bone in childhood. Less frequently it occurs in soft tissues [1].

Ewing's sarcoma/primitive neuroectodermal tumor (ES/PNET) is an extraordinarily rare primary tumor in the kidney. Only few cases of primary renal Ewing's sarcoma have been reported in the literature to date [1–7]. Renal localization of pPNETs is found in young adults and is characterized by an aggressive clinical course and poor prognosis [1].

We present here two cases of Ewing's sarcoma/primitive neuroectodermal tumor occurring in two young women with an unusual presentation. The diagnosis in both cases was incidental during visits to the emergency room for other health problems.

## 2. Case Report

### 2.1. Case 1

**Figure 1:**
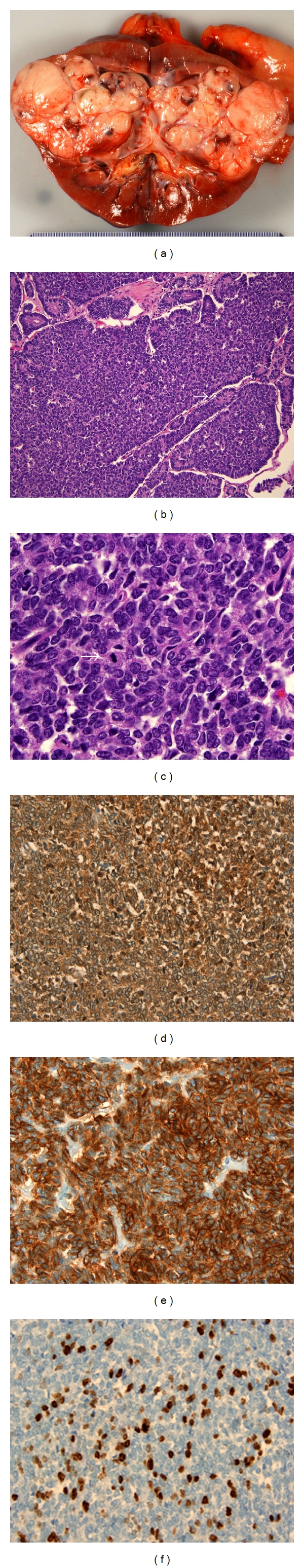
Thirty-two-year-old patient, right-sided laparoscopic radical nephrectomy. (a) Gross picture of the right kidney showing an 8.3 cm encapsulated tumor located at the pelvic adipose tissue within the renal capsule. (b) Microscopic photography of the tumor showing tumor cells arranged in solid sheets, tightly packed cords, and trabeculae with variable stroma. Tubular structures (arrow) can be identified in the periphery of the cell nests (HE ×20). (c) High power view of the tumor cells showing uniform small round blue cells with scant cytoplasm, uniform nuclei, and stippled chromatin. Mitotic activity (arrow) was present (HE ×100, oil). (d) The tumor cells were strongly positive for NSE in a cytoplasmic stain (DAB X50). (e) Strong membranous and cytoplasmic stain for CD56 (DAB X50). (f) Ki 67 shows a high proliferation rate index of 30% (DAB X50).

A 32-year-old female presented to the emergency room with right flank pain that irradiates to the groin. She was clinically diagnostic with kidney stones and indeed a kidney stone was identified. However, the computed tomography scan revealed a large right renal mass and right ovarian cystic mass. The patient underwent a right-sided laparoscopic radical nephrectomy along with an excision of the ovarian cystic mass. Her postoperatively course was uneventful except for some pain in the surgical incision site. Grossly, an 11 cm cyst adenofibroma of the right fallopian tube and ovary was identified. In addition, the kidney showed an 8.3 cm encapsulated tumor located at the pelvic adipose tissue within the renal capsule (Figure 1(a)). Microscopically, the tumor cells were arranged in solid sheets, tightly packed cords, trabeculae with variable stroma, and nests or ribbons of uniform small round blue cells with scant cytoplasm, uniform nuclei, and stippled chromatin. At places rosettes were identified (Figure 1(b)). Mitotic activity was present (Figure 1(c)). No necrosis was seen. Immunohistochemical stains were performed on formalin-fixed, paraffin-embedded tissue using the usual avidin-biotin-peroxidase complex method. The antibodies are listed in Table 1. The tumor cells were strong positive for neuron-specific enolase (Figure 1(d)), CD56 (Figure 1(e)), Cytokeratin AE1/3, and Cam 5.2. Weakly positive stain for PGP9.5, bcl2, and CD99 was identified. The tumor was negative for synaptophysin, EMA, inhibin, chromogranin, vimentin, WT1, S100, CK7, estrogen receptor, androgen receptor, CD10, and Carbonic anhydrase 9. Ki 67 showed a high proliferation rate index of 30% (Figure 1(f)). Fluorescent in situ hybridization analysis using the 22q12 LSI, EWSR1, Dual-Color Break-Apart Probe was performed on fresh tissue and showed 18 (29.5%) of the cells with the translocation pattern 22q12 for the EWSR1 gene. The histomorphological, immunohistochemical profile and FISH results were consisted with a renal primitive neuroectodermal tumor/EWING tumor. The patient underwent chemotherapy and the followup 2 years after the diagnosis showed no evidence of tumor.

### 2.2. Case 2

**Figure 2:**
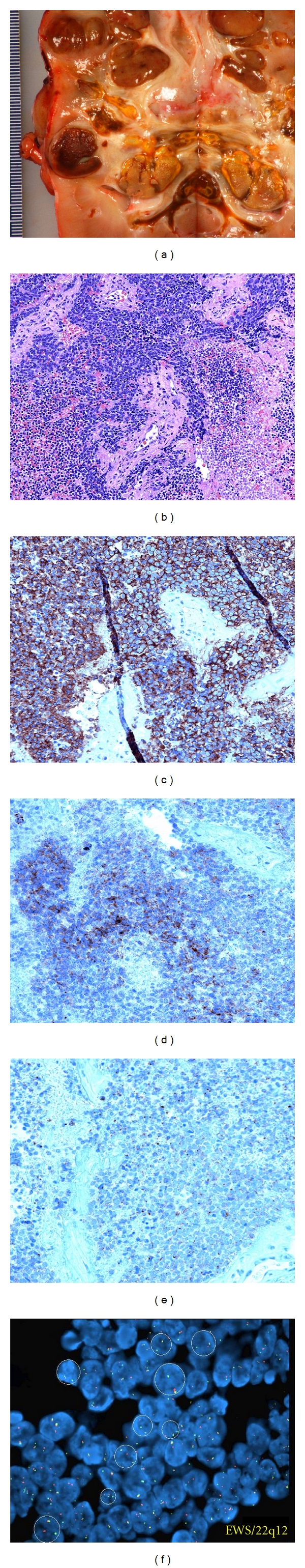
Twenty-one-year-old female. (a) Bivalved right kidney: gross photography showing islands of tan tissue with hemorrhage and necrotic yellow friable areas (center). (b) Microphotography of the right atrial mass removed from the patient showing islands of small blue cells intermixed with necrotic material and blood. (c) The tumor cells showed positive membranous satins for CD99 (DAB X40). (d) Viable tumor cells positive for CD56, note that the necrotic cells are negative (DAB X40). (e) Synapthophysin was positive in the viable neoplastic cells in a cytoplasm dot-like pattern (DAB X40). (f) Fluorescent microphotography: normal cell lacking t(22; q12), a 2-fusion signal pattern is expected to be seen, reflecting the 2 intact copies of EWSR1. In abnormal cell (circle) with t(22; q12), a 1-fusion, 1 green, 1 orange signal pattern will be expected to occur. This confirmed the EWSR1 fusion transcripts in this peripheral primitive neuroectodermal tumor of the kidney.

A 21-year-old female presented to the emergency room with a left foot fracture, nasal fracture, and loss of her pregnancy caused by a motor vehicle accident. A month later, the patient returned to the emergency room with complaints of shortness of breath, icterus, and dark urine. A chest-computed tomography scan revealed pulmonary embolism. She underwent a pulmonary embolism protocol which included a computed tomography of the chest, abdomen, and pelvis. The exam showed extensive central bilateral pulmonary embolism and a large right atrial thrombus in continuity with thrombotic material within the inferior vena cava. In addition, her right kidney had poorly contrast-enhancing raising the possibility of a right renal vein thrombosis extending into the right atrium with subsequent pulmonary embolism. She underwent a pulmonary tumor embolectomy, right atrial mass removal, and tumor embolectomy thrombectomy of the hepatic inferior vena cava as well as placement of an inferior vena cava filter. The patient underwent a sternotomy with cardiopulmonary bypass. She started chemotherapy with etoposide and ifosfamide combination therapy. The tumor invaded the renal vein and extended to the resection margin. The histological examination of the right atrial, pulmonary artery, and caval vein thrombus revealed islands of uniform small round blue cells with scant cytoplasm, uniform nuclei, and stippled chromatin intermixed with necrosis and blood clot (Figure 2(b)). The viable tumor cells showed brisk mitosis. Immunohistochemical stains were performed on formalin-fixed, paraffin-embedded tissue using the usual avidin-biotin-peroxidase complex method. The antibodies are listed in Table 1. The viable tumor cells showed a strong membranous stains for CD99 (Figure 2(c)), focal membranous and cytoplasm stain for CD56 (Figure 2(d)), a cytoplasm dot pattern for synapthophysin (Figure 2(e)), and focal cytoplasm and membranous stain for PGP9.5, BCL2, and vimentin. The lymphoid cells in the background were positive for LCA and CD43. The tumor cells are negative for CD3, CD20, CD30, alpha-fetoprotein, beta human chorionic gonadotrophin, pan-cytokeratin, CAM5.2, myogenin, epithelial membrane antigen, thyroid transcription factor-1, CK20, desmin, smooth muscle actin, HMB45, S100, and WT1. Five months later, the patient had a right nephrectomy. Grossly, the right kidney revealed multifocal tan-brown to yellow masses in the upper and lower poles ranging from 0.4 × 0.4 cm to 4.3 × 2.8 cm (Figure 2(a)). Fluorescent in situ hybridization analysis using the 22q12 LSI, EWSR1, Dual-Color Break-Apart Probe was performed on fresh tissue and showed 57 (95.0%) of the cells with the translocation pattern 22q12 for the EWSR1 gene (Figure 2(f)). In addition a FISH for the X-18 SYT translocation was performed but it was negative. The histomorphological, immunohistochemical profile and FISH results were consisted with a renal primitive neuroectodermal tumor/EWING tumor. The patient completed her 12 months of chemotherapy and the one-year followup revealed no evidence of tumor.

## 3. Discussion

The diagnosis of renal primitive neuroectodermal tumor/EWING tumor is very rare and usually involves several different diagnostic techniques. The most challenging part is the differential diagnosis which is broad with morphological and immunohistochemical overlapping features between the entities. Therefore, mostly frequently molecular biology techniques need to be applied for a definitive diagnosis. We reported 2 cases of renal primitive neuroectodermal tumor/EWING tumor with an unusual clinical presentation which were incidentally found during imaging. These cases illustrated very well the difficulties with which general pathologists are faced in the routine anatomical pathology examination when faced with renal poorly differentiated neoplasm in adults.

Both cases had an uncanny presentation. In case number 1, the 8.3 cm kidney mass was discovered after the patient presented clinically with irradiating flank pain, mimicking the pain related with kidney stones. Moreover, it was associated with a benign ovarian tumor and the diagnosis of renal primitive neuroectodermal tumor/EWING was only possible after extensive immunohistochemical and molecular studies, which were mainly performed to rule out other entities that were considered due to the unusual presentation and associated ovarian tumor. In literature-reported renal primitive neuroectodermal tumor/EWING, the clinical presentation was often nonspecific, comprising abdominal pain, palpable mass, and hematuria, and most reported cases were diagnosed incidentally by image studies [1, 4–8]. Few tumors had associated concurrent abnormalities such as hemangioma, central nervous system primitive neuroectodermal tumor, and ovarian disgerminoma [1].

Our second case report was even more unusual. The Case 2 presented with respiratory problems after being involved in an automobilistic accident where multiple fractures were acquired. The clinical presentation suggested pulmonary thormboembolism, which was confirmed by image; however, after histopathological examination of the thrombectomy material small blue cell tumor of uncertain origin was regarded as the cause for the extensive thromboembolic event. Later on, a nephrectomy was performed and a renal primitive neuroectodermal tumor/EWING was diagnosed. As far as literature reports, there are few reports of renal primitive neuroectodermal tumor/EWING with tumor thrombus extending to the inferior vena cava [9, 10]. However, in our case the thrombus extended all the way up to the inferior vena cava, right atrium, and bilateral pulmonary arteries, with more aggressive tumor extension than the previous reported cases [9, 10].

As far as disease progression, our cases had a better response to chemotherapy and surgery than the previous reported cases of renal primitive neuroectodermal tumor/EWING [1]. The outcome of other patients reported in the literature included very short survival, nonresponse to intensive therapy, and death 5 months after diagnosis [1, 4–8, 11]. Both our cases were free of disease after one year, and long-term outcome is still awaited.

Renal primitive neuroectodermal tumor/EWING tumor should be differentiated from other small blue cell tumors arising in the kidney such as blastemal predominant Wilms tumor, neuroblastoma, rhabdomyosarcoma, lymphoblastic lymphoma, small cell neuroendocrine carcinoma, desmoplastic small blue cell tumor, and synovial sarcoma [1, 12]. All of them have very similar morphology with frequent overlapping morphological features, thus, extensive immunohistochemistry panel, in addition to the morphological features, is essential in reaching the final diagnosis [1, 12].

CD99 was the former marker for Ewings' sarcoma; however, it is also expressed in other tumors such as synovial sarcoma, Wilms' tumor, vascular malignancy, neuroendocrine tumors, and lymphoblastic lymphoma, and therefore nowadays is not considered a reliable marker of tumor origin [1, 7, 8]. Our presented first case showed a very weak stain for CD99, and the second one because of the bloody background was very difficult to interpret illustrating some of the difficulties in using this marker in diagnostic grounds.

From the neuroendocrine marker, CD56 was positive in both cases whereas synapthophysin was positive only in the second case in a cytoplasm dot pattern. This pattern has been considered as an unspecific pattern, but a recent publication described the pattern in oligodendrogliomas, and therefore the pattern seems to indicate neural origin [13].

The features favoring the diagnosis of blastemal Wilms tumor are young age and positivity of blastemal elements for vimentin, low molecular weight cytokeratin, epithelial membrane antigen, and WT1. Both cases in our study were positive for cytokeratin and vimentin. However, WT1 was negative and EWSR1 gene was identified by FISH, and therefore adult Wilms' tumor was ruled out.

Renal neuroblastomas are also similar morphologically to renal primitive neuroectodermal tumor/EWING tumor. However, they usually occur at the age of 5 years and show a fibrillary background or a ganglionic differentiation facilitating the diagnosis. Immunohistochemistry is very useful to differentiate it from renal primitive neuroectodermal tumor/EWING tumor because neuroblastomas are consistently positive for NSE and chromogranin and mostly negative for CD99.

Rhabdomyosarcoma is often positive for smooth muscle actin, desmin, and myogenin; all of them were negative in our patients. Desmoplastic small blue cell tumors are also positive for desmin, and synovial sarcoma was ruled out using the FISH for the X-18 SYT translocation, which was negative.

Finally, lymphoblastic lymphoma could be excluded by performing analysis with LCA and other hematopoietic markers; however, in the second case the background was positive for lymphoid markers. The differential was performed by morphology of the tumor cells which had stippled chromatin and pleomorphism whereas the lymphoid cells formed a uniform monotonous population with small dark nuclei in the background.

In our cases, the molecular analysis identifying the translocation was a supportive and strong confirmatory tool, especially with the confusing immunohistochemical profile. Fluorescent in situ hybridization analysis using the 22q12 LSI, EWSR1, and Dual-Color Break-Apart Probe showed the translocation pattern 22q12 for the EWSR1 gene. The probe used in our cases is from Vysis (Abbott Laboratories, Downers Grove, Ill, USA) and is commercially available, and data have been published using this probe. The probe is an EWSR1 (22; q12) Dual-Color, Break-Apart Rearrangement Probe, which consists of a mixture of 2 FISH DNA probes. The first is a 500 kb probe labeled in spectrum orange and flanks the 5 V side of EWSR1 gene and extends inward into intron 4. The second probe is 1100 kb labeled in spectrum green and flanks the 3 V side of the EWSR1 gene. There is 7-kb probe between the 2 probes. The known breakpoints within the EWSR1 gene are restricted to introns 7 through 10V. Approximately 90% of the translocations involving EWSR1 gene result in t(11; 22) translocation characteristic of EWS/PNET.

Because most renal primitive neuroectodermal tumors/EWING tumor of the kidney are poorly differentiated and have a broad morphological spectrum, it is important to have genetic confirmation for the presence of t(11; 22) for the accurate identification of these tumors, and the FISH probe is a good and easy method to do so.

In summary, we report two cases of primitive neuroectodermal tumor/EWING tumor arising in the kidney confirmed by morphology, immunohistochemistry and FISH. The unusual presentation in our cases delayed the diagnosis, being both tumors diagnosed incidentally. Thus, we believe that these cases added some features to the already broad spectrum of presentations described in renal primitive neuroectodermal tumor/EWING tumor and also showed the importance of molecular analysis together with morphological and immunohistochemical features for confirmation of this poorly differentiated neoplasm.

## Figures and Tables

**Table 1:** Specifications of the antibodies used in the study.

Antibody	Clone	Company	Dilution	Retrieval
NSE	O10	Immunotech (France)	PD	CC1 mild 30”
CD56	7	Vector (US)	1/10	CC1 mild 30”
CKAE1/3	PGM-1	DAKO (Denmark)	1/100	CC1 mild 30”
Cam 5.2	KP-1	VENTANA (USA)	PD	CC1 mild 30”
PGP9.5	10D6	VECTOR (UK)	1/250	CC1 mild 30”
Bcl2	Polyclonal	NOVACASTRA (UK),	1/500	None
CD99	12D6	VECTOR (UK)	1/100	CC1 mild 30”
Synaptophysin	Polyclonal	GENETEX, Inc. (USA)	1/25	Protease digestion 4”
EMA	55K-2	DAKO (Denmark)	1/500	CC1 mild 30”
Ki67	MIB-1	DAKO (Denmark)	1/25	CC1 mild 30”
Inhibin	RI'	DAKO (Denmark)	1 : 50	CC1 standard 60”
Chromogranin	LK2H10	VENTAN (USA)	PD	None
Vimentin	V9	VENTANA (USA)	PD	CC1 standard 60”
WT1	6F-H2	DAKO (Denmark)	1 : 25	CC1 standard 60”
S100	Polyclonal	DAKO (Denmark)	1 : 500	CC1 short 8”
CK7		DAKO (Denmark)	1 : 200	Protease 24”
ER	SP1	VENTANA (USA)	PD	CC1 standard 60”
Pan-CK		ABCAM (USA)	1 : 100	CC1 mild 30”
CAM5.2	CAM5.2	Beckton-Dickison (USA)	1 : 10	Protease 18”
Myogenin		VENTANA (USA)	PD	CC1 standard 60”
TTF1	B72.3	BIOGENEX (USA)	1 : 50	CC1 mild 30”
CK20	K5208	DAKO (Denmark)	1 : 50	CC1 mild 30”
BHCG		VENTANA	PD	CC1 mild 30”
AFP	Polyclonal	DAKO (Denmark)	1 : 2000	CC1 standard 60”
CD30	BER-H2	VENTANA (USA)	PD	CC1 standard 60”
CD20	L26	VENTANA (USA)	PD	CC1 mild 30”
CD3	Polyclonal	DAKO (Denmark)	1 : 100	CC1 mild 30”
CD43	L60	VENTANA (USA)	PD	CC1 short 8”
LCA	RP2/18	VENTANA (USA)	PD	CC1 mild 30”
CA9	Polyclonal	NOVUS BIOLOGICALS (USA)	1 : 1000	CC1 mild 30”
CD10		CELL MARQUE (USA)	PD	CC1 standard 60”
AR	AR441	DAKO (Denmark)	1 : 100	CC1 standard 60”
Desmin	DE-R-11	VENTANA (USA)	PD	CC1 mild 30”
SMA	IA4	VENTANA (USA)	PD	None
HMB45	CMA710	CELL MARQUE (USA)	PD	CC1 mild 30”

CC1: Cell Conditioning 1 is antigen retrieval from Ventana Benchmark similar to high pH EDTA buffer. PD: prediluted, CA9: carbonic anhydrase 9, SMA: smooth muscle actin, AR: Androgen receptor, AFP: alpha-fetoprotein, TTF1: thyroid transcription factor-1, ER: estrogen receptor, and NSE: neuron-specific enolase.
